# A trait-based ecology to assess the acclimation of a sperm-dependent clonal fish compared to its sexual host

**DOI:** 10.7717/peerj.5896

**Published:** 2018-11-02

**Authors:** Christelle Leung, Sophie Breton, Bernard Angers

**Affiliations:** Department of Biological Sciences, Université de Montréal, Montréal, QC, Canada

**Keywords:** Sexual and asexual organisms, Phenotypic plasticity, Trophic niche, Gynogenesis, *Chrosomus eos-neogaeus* complex, Geometric morphometrics, Hybrids, Common garden, Trait-based ecology

## Abstract

**Background:**

Survival in temporally or spatially changing environments is a prerequisite for the perpetuation of a given species. In addition to genetic variation, the role of epigenetic processes is crucial in the persistence of organisms. For instance, mechanisms such as developmental flexibility enable the adjustment of the phenotype of a given individual to changing conditions throughout its development. However, the extent of factors other than genetic variability, like epigenetic processes, in the production of alternative phenotype and the consequences in realized ecological niches is still unclear.

**Methods:**

In this study, we compared the extent of realized niches between asexual and sexual individuals from different environments. We used a trait-based ecology approach exploiting trophic and locomotive structures to infer the environment that each biotype actually used. More specifically, we compared the morphology of the all-female clonal and sperm-dependent fish *Chrosomus eos-neogaeus* to that of their sexual host species *C. eos* in common garden and natural conditions.

**Results:**

Transfer from natural to controlled conditions resulted in a similar shift in measured morphology for clonal and sexual individuals suggesting comparable level of flexibility in both kinds of organisms. However, clonal, but not sexual, individuals displayed a consistent phenotype when reared in uniform conditions indicating that in absence of genetic variation, one phenotype corresponds to one niche. This contrasted with results from natural conditions where clones were morphologically as variable as sexual individuals within a sampled site. In addition, similar phenotypic changes for both clonal and sexual individuals were observed among the majority of sampled sites, indicating that they responded similarly to the same environments.

**Discussion:**

Our results indicated that clones can efficiently use different niches and may evolve in a range of environmental conditions comparable to that of a sexual species, thus underlying the importance of factors other than genetic variability, like epigenetic processes, for coping with environmental heterogeneity.

## Introduction

Understanding the different factors underlying the development of phenotypic variation is of major importance in ecological and evolutionary biology, as such phenotypic variation enables organisms to survive and reproduce successfully in heterogeneous and fluctuating environments (Agrawal, 2001a; Price, Qvarnström & Irwin, 2003). Genetic variability is responsible for the production of variable phenotypes, and it has long been recognized as the main factor allowing the organisms to thrive in heterogeneous environmental conditions (Barton & Charlesworth, 1998; Crow, 1994; Lande & Shannon, 1996; Reed & Frankham, 2003). Sexual reproduction can introduce new gene combinations and is therefore an effective means for producing genetic variation (Drake et al., 1998; Kondrashov, 1988). However, sexually reproducing organisms have to deal with different short-term costs (Agrawal, 2001b; Bell, 1982; West & Peters, 2000; Williams, 1975), including the breakdown of adaptive gene combinations that results in lower average fitness of sexual progeny, and the production of males which entails a demographic cost, that is, the “twofold cost of sex” (Maynard Smith, 1978). Furthermore, genetic variation alone is not enough to explain the persistence of organisms in highly fluctuating environments (Chevin, Lande & Mace, 2010; Lande & Shannon, 1996), neither is the widespread distribution of some genetically identical organisms (Lynch, 1984; Neiman, Meirmans & Meirmans, 2009; Vrijenhoek, 1998). Individuals expressing the phenotype that provides the maximum fitness within a given environment should be favored by natural selection. As a consequence, local adaptation should increase adaptive allele frequencies in the population (Barrett & Schluter, 2008; Savolainen, Lascoux & Merilä, 2013). However, when environmental changes are too frequent over time, the selected phenotype may not be optimal anymore, suggesting that alternative strategies for generating phenotypic variation more quickly will be promoted (Burger & Lynch, 1995).

It has been shown that epigenetic processes can result in phenotypic differences among individuals, even in the absence of genetic variation (Jaenisch & Bird, 2003; Kucharski et al., 2008; Matsumoto et al., 2013). Phenotypic variation can arise from two epigenetic sources underlying different ecological strategies: environmentally induced epigenetic variation and stochastic epimutations, which have been proposed to be among the potential mechanisms underlying phenotypic plasticity and diversifying bet hedging strategies, respectively (Castonguay & Angers, 2012; Leung, Breton & Angers, 2016; Piggot, 2010; Vogt, 2015). Epigenetic processes are thus particularly important for asexual organisms, which were often defined as “evolutionary dead-ends” because of the long-term costs associated to asexual reproduction, namely the limited ability to adapt to changing environments and the accumulation of deleterious mutations (Mayr, 1970; Müller, 1964; Maynard Smith, 1978; Stalker, 1956; Uzzell, 1970). Asexual organisms can rely on different strategies to cope with environmental changes. For example, the general-purpose genotype model (Baker, 1965) involves a generalist genotype that is capable of tolerating a broad range of environmental conditions. Such flexibility of a genotype would allow a given clonal lineage to cope with spatially and temporally heterogeneous environments (Lynch, 1984), but without the different costs associated to sexual reproduction (Agrawal, 2001b; Bell, 1982; West & Peters, 2000; Williams, 1975).

Given the different sources of phenotypic variation, both sexual and asexual organisms have the potential to cope with environmental heterogeneity. The relative importance of phenotypic plasticity and genetic variation to cope with varying environmental conditions remains, however, not well understood as the different strategies encompass different costs. For instance, while phenotypic plasticity allows a given genotype to cope with environmental variation, it can also be associated to different costs and limits, including energetic costs in the maintenance and production of different phenotypes, developmental instability due to greater susceptibility of errors in the production of alternative phenotypes, or the reliability of environmental signals (Bradshaw, 1965; DeWitt, Sih & Wilson, 1998; Leung et al., 2017; Murren et al., 2015; Pigliucci, 2001). The extent of alternatives to genetics, such as epigenetic processes, in the production of different phenotypes and the consequences in realized ecological niches remain therefore unclear.

In this study, we used a trait-based ecology approach to compare the extent of the realized niches between asexual and sexual individuals from different environments. Morphological characteristics are known to be a reliable indicator of the ecological niche in fishes, like mouth shape and size for trophic niche (Karpouzi & Stergiou, 2003; Keast & Webb, 1966; Spreitzer et al., 2012) or locomotor-associated traits for habitat conditions such as water velocity (Laporte et al., 2016; Magnan et al., 2014). We compared the morphology of the all-female clonal sperm-dependant fish *Chrosomus eos-neogaeus* to that of its sexual host species *C. eos* in common garden and natural conditions. Clonal sperm-dependent systems represent a suitable framework to test the relative importance of phenotypic plasticity and genetic variation to cope with varying environmental conditions because these clonal organisms are tightly linked to their sexual hosts in natural populations. In gynogenetic systems, also known as sperm-dependent parthenogenesis, clonal individuals benefit from the demographic advantage of asexuality, but they cannot outcompete the related sexual species as they rely on their sperm to trigger embryogenesis (Beukeboom & Vrijenhoek, 1998; Hubbs, 1964; Schlupp, 2005; Vrijenhoek, 1998). Thus, the coexistence of both clonal and sexual sperm-donor species is obligatory in such systems, resulting in a complex ecological dynamic. Indeed, the demographic advantage of asexual organisms could result in the extinction of the sexual hosts and ultimately lead to their own demise due to the lack of sperm source (Kokko, Heubel & Rankin, 2008; Lehtonen et al., 2013; Leung & Angers, 2018). Furthermore, despite the lack of genetic variability among individuals, clonal individuals have to cope with the same environmental heterogeneity than their sexual counterparts.

We first compared the extent of phenotypic plasticity as a short-term response to environmental changes, for clonal individuals and their sexual parental species by performing common garden experiments. If both asexual and sexual biotypes exhibit similar levels of phenotypic plasticity, then we would expect to observed similar morphological changes for both biotypes following their transfer from natural to experimental environments. However, rearing individuals in homogeneous conditions will minimize the environmental effect on phenotypic variation. Therefore, a group of genetically identical individuals (clonal biotype) is predicted to be less variable than a group of genetically variable individuals (sexual biotype) because of the genetic influence on phenotypic variation.

We thereafter compared the morphological variation of both sexual and asexual individuals in natural environments. If alternatives to genetic variation enable a clonal lineage to thrive as well as sexual organisms in the same heterogeneous environment, both biotypes are expected to display similar degree of morphological differences according to contrasting environmental conditions. Furthermore, if both asexual and sexual individuals occupied the same ecological niches, we expect, as in common garden experiments, that asexual biotype will be less variable than sexual one because of the genetic influence on phenotypic variation.

## Materials and Methods

### Study system

Gynogenetic *C. eos-neogaeus* originated from multiple hybridization events between the redbelly dace *C. eos* and the finescale dace *C. neogaeus* (Dawley, Schultz & Goddard, 1987; Goddard et al., 1998; Goddard & Dawley, 1990). Asexual *C. eos-neogaeus* are found in various types of environments, such as ponds, streams, or lakes (Leung, Breton & Angers, 2016; Schlosser et al., 1998; Scott & Crossman, 1973), and, as they are sperm-dependent, they co-occur with at least one of the parental species.

The capacity of *C. eos-neogaeus* clones to adjust their phenotype according to environmental conditions was highlighted by recent studies that reported distinct epigenetic profiles according to environmental conditions (Leung, Breton & Angers, 2016; Massicotte & Angers, 2012), although those studies did not attempt to link epigenetic variation to phenotypic traits. In natural conditions, *C. eos-neogaeus* clones were, however, found to be phenotypically as variable as their sexual sperm-donor (Doeringsfeld et al., 2004), suggesting that they may cope with environmental heterogeneity with as much phenotypic variation than their genetically variable parental species. Still, the use of several discriminant traits (such as the number of pharyngeal teeth or intestinal loops) revealed no overlap between clones and *C. eos* individuals’ morphology (Doeringsfeld et al., 2004), hampering the assessment of whether both biotypes use the same ecological niches. However, since both sexual and asexual species relied on different processes to develop a given phenotype, it appears crucial to determine whether or not the two species occupy the same ecological niche. If the morphology of both species overlaps, it could mean they converged toward the same morphological optimum to occupy the same niche, but by using distinct processes. Otherwise, it is not possible to distinguish between “they display different phenotypes because they did not occupy the same niche” or “they occupy the same niche but one of the species did not develop an optimal phenotype.”

### Sampling and genetic identification

Two regions in southern Quebec (Canada) that display two contrasting distribution of different lineages were surveyed in this study. The Western region is characterized by the dominance of a single widespread hybrid lineage, whereas the presence of multiple distinct hybrid lineages within one drainage basin was reported in the Eastern region (Leung, Breton & Angers, 2016; Vergilino, Leung & Angers, 2016). These two regions are also characterized by the scarcity of the parental species *C. neogaeus*, making the parental species *C. eos* the principal sperm-donor of *C. eos-neogaeus* (Angers & Schlosser, 2007; Vergilino, Leung & Angers, 2016).

**Figure 1:**
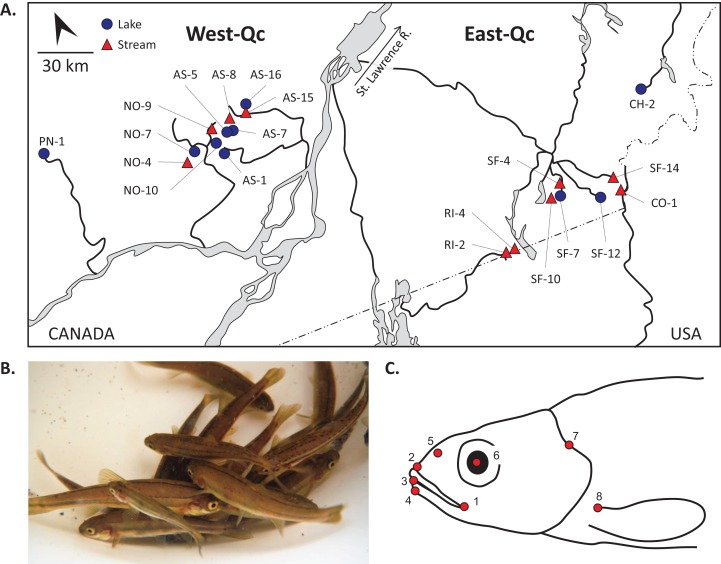
Fish and landmark sampling. (A) Map of the southern Quebec (Canada) and geographic location of sampling sites. Codes for lake (blue circles) and stream (red triangles) are according to Vergilino, Leung & Angers (2016). (B) Individuals of the complex *Chrosomus eos-neogaeus*. Photograph by Christelle Leung. (C) Schematic positions of the eight landmarks. The shape of the head was assessed with landmarks around the mouth (1–4), the posterior part of the nostril (5), the middle of the eye (6), the dorsal peak of the operculum (7), and the insertion of the most dorsal pelvic spin (8).

We selected a total of 20 localities (Fig. 1A; Table 1) to sample *Chrosomus* spp. fishes. Because high- and low-velocity habitat conditions are known to influence fish morphology (Berner et al., 2008; Collin & Fumagalli, 2011; McGuigan et al., 2003; Senay, Boisclair & Peres-Neto, 2015), 10 small lakes or ponds and 10 streams were selected in order to maximize phenotypic differences according to contrasting environmental conditions in natural populations. Adult fishes from the different sampling sites were captured passively using minnow traps. Distinction between *C. eos-neogaeus* and *C. eos* was performed a posteriori using genetic tools.

**Table 1:** Characteristics of sampled sites in natural condition.

Region	Habitat type	Hybrid lineages	Site	Geographic coordinates	Sample size		
					*C. eos*	Hybrids	Total
West-QC	Lake	B-01	AS-1	N 45°55′00.9″ W 74°04′22.5″	12	19	31
West-QC	Lake	B-01	AS-16	N 46°05′35.3″ W 73°52′15.5″	19	35	54
West-QC	Lake	B-01	AS-7	N 45°59′23.5″ W 74°00′00.0″	11	17	28
West-QC	Lake	B-01	NO-7	N 45°56′37.6″ W 74°11′37.7″	0	13	13
West-QC	Stream	B-01	AS-15	N 46°05′34.6″ W 73°52′20.4″	0	10	10
West-QC	Stream	B-01	AS-8	N 46°05′20.8″ W 73°57′21.8″	0	28	28
West-QC	Stream	B-01	NO-4	N 45°54′16.3″ W 74°18′46.0″	0	16	16
West-QC	Stream	B-01	NO-9	N 45°56′04.3″ W 74°06′11.3″	0	29	29
West-QC	Lake	–	AS-5	N 45°59′17.1″ W 74°00′24.7″	32	0	32
West-QC	Lake	–	NO-10	N 45°55′32.4″ W 74°03′51.1″	20	0	20
West-QC	Lake	–	PN-1	N 46°12′43.0″ W 75°13′60.0″	23	0	23
East-QC	Stream	A-06	CO-1	N 45°09′15.1″ W 71°32′53.3″	16	17	33
East-QC	Stream	A-06	SF-14	N 45°11′04.5″ W 71°33′13.2″	13	35	48
East-QC	Stream	A-11	RI-2	N 45°02′35.8″ W 72°21′43.1″	16	17	33
East-QC	Stream	A-11	RI-4	N 45°03′01.4″ W 72°19′03.3″	16	10	26
East-QC	Lake	A-18	SF-12	N 45°07′48.5″ W 71°40′21.9″	22	31	53
East-QC	Stream	B-06	SF-4	N 45°14′01.8″ W 71°54′28.0″	18	19	37
East-QC	Lake	B-06	SF-7	N 45°12′56.7″ W 71°54′31.5″	22	16	38
East-QC	Lake	–	CH-2	N 45°29′03.7″ W 71°04′48.3″	20	0	20
East-QC	Stream	–	SF-10	N 45°12′37.3″ W 71°56′10.2″	30	0	30
				Total	290	312	602

**Note:**

Site and lineage codes are according to Vergilino, Leung & Angers (2016).

Total DNA from the caudal fin of each individual was extracted according to Sambrook, Fritsch & Maniatis (1989). Genetic identification of individuals was performed according to Binet & Angers (2005) to discriminate the different biotypes and according to Vergilino, Leung & Angers (2016) to identify the different hybrid lineages. As the hybrid biotype reproduces clonally, individuals of a given lineage are expected to be genetically identical, whereas genetic differences are observed among lineages (Vergilino, Leung & Angers, 2016). The presence of several lineages per site was reported for the sampling sites selected for this study (Vergilino, Leung & Angers, 2016). However, because genetic variation among lineages could represent confounding variation explained by environments, we restricted the morphological analyses to five distinct lineages (genotypes) within hybrid individuals, that is, one lineage per sampled site (Table 1).

### Common garden experiments

We transferred individuals from natural environments to controlled conditions to determine whether individuals respond to a uniform environment with a consistent morphology, as expected for plastic individuals confronted to a given environmental condition (Debat & David, 2001). Common garden experiments were performed to provide a stable and homogeneous environment in aquarium: luminosity was set up according to natural photoperiod, that is, lengths of light exposure in a 24-h period similar to natural conditions in southern Quebec, temperature was constant at 19 °C, oxygen was maintained at saturation level and individuals were fed ad libitum. A single trophic niche was mimicked by feeding individuals with floating micro-pellets fish food, constraining them to reach the surface of the water to eat. Similarly, locomotion was expected to change within an aquarium with reduced water flow at the opposite of natural environments. Larvae (<1 cm) were sampled from one lake (*n* = 24; site AS-16 N 46°05′35.5″ W 73°52′15.7″) and one stream (*n* = 39; site RI-2 N 45°02′35.8″ W 72°21′43.1″). It has been shown that morphological changes can occur rapidly in controlled conditions (Laporte et al., 2016; Proulx & Magnan, 2004); thus, experimental conditions were maintained for 5 months. At the end of the experiment, individuals reached adult size (≈5 cm, similar to individuals from natural environments), and were sacrificed and genetically identified according to the procedure described above.

Sampling and common garden experiments were performed under institutional animal care guidelines (permit #13-084 delivered by the *Université de Montréal*), and conform to the mandatory guidelines of the Canadian Council on Animal Care. Sampling permits were provided by the Quebec Ministry of Natural Resources and Wildlife (MRNF; permit #2012-09-11-124-05-S-P, #2013-09-17-128-05-S-P and #2014-07-25-1105-15-SP).

### Morphological measurement

Given the link between mouth morphology and trophic niche (Carlson & Wainwright, 2010; Gerry et al., 2013; Keast & Webb, 1966; Langerhans et al., 2003), the shape of the head was measured on individuals reared in common gardens and those sampled from natural localities. Geometric morphometrics (Adams, Rohlf & Slice, 2004; Klingenberg, 2010; Mitteroecker & Gunz, 2009; Zelditch, Swiderski & Sheets, 2012) was used to quantify shape variation of individuals based on a two-dimensional system. Close-up of the left side of the head was digitized using macro photography with the camera lens positioned parallel to the plane of the individual in lateral view, and eight homologous landmarks (Fig. 1C) were positioned on digitized pictures using Tpsdig2 software (Rohlf, 2010).

### Statistical analyses

The configurations of landmarks were subjected to a Generalized Procrustes Analysis that standardizes and rotates landmarks coordinates. This analysis allowed to rule out any information not related to intrinsic form; that is, size, position and orientation of individuals relative to picture digitization (Gower, 1975; Mitteroecker & Gunz, 2009; Rohlf, 1999; Rohlf & Slice, 1990). Shape variables were thereafter extracted from the resulting aligned Procrustes coordinates projected to the shape-tangent space (Dryden & Mardia, 1993; Rohlf, 1999) before their use for subsequent multivariate analyses.

#### Measurement error

To estimate measurement error due to parallax in 2D imaging, a subsample of 100 individuals randomly chosen from natural populations were digitized twice by different operators. Similarly, we estimated landmark positioning error by placing landmarks twice for a subsample of 100 pictures randomly chosen from a given digitizing session. The relative amount of shape variation attributable to image digitizing or landmark positioning were assessed using a Procrustes analysis of variance (ANOVA) analysis (Klingenberg & McIntyre, 1998) and significance were tested with permutation tests using 999 randomizations.

#### Genetic and environmental effect on shape variation

We used partial redundancy analyses (Borcard, Legendre & Drapeau, 1992) to assess the genetic and environmental effects on the total shape variation. We used the shape variables of the resulting aligned Procrustes coordinates as the response variable. For the explanatory variable, we identified each individual according to their biotype (sexual vs asexual) and the environment they came from (common garden vs sampled localities). Because genetic variation influence the development of phenotypic variation and every hybrid lineages were not found in all sampling sites, we also grouped sampling sites harboring the same clonal genotype together. Thereafter, we used those groups as a conditioning matrix (i.e., matrix containing the variables whose effects are to be partialled out) in subsequent partition of variation analyses to control for the genetic differences that exist among the distinct clonal lineages as well as for the sexual *C. eos* species at those sites. Finally, we coded all the explanatory factors and their interaction with orthogonal dummy variables obtained by Helmert contrast (Legendre & Anderson, 1999; Legendre & Legendre, 1998). The percentages of the total shape variation that can be attributed to biotypes and environments were based on the adjusted *R*^2^ (*R*_a_^2^) (Peres-Neto et al., 2006) and significance of each *R*^2^ was tested by permutation tests using 999 randomizations.

We used phenotypic trajectory analyses (Adams & Collyer, 2009; Collyer & Adams, 2007, 2013) to compare the magnitudes and direction of shape changes due to the transfer from natural to controlled condition for clonal and sexual individuals, as well as to compare the magnitude of within-site shape differences between sexual and asexual individuals among different sampling sites.

We also assessed how the shape of asexual individuals changed among sampling sites compared to that of sexual individuals coping with the same environments. To do so, we tested the concordance of their respective morphospace for sites where they were found in sympatry. First, principal component analysis (PCA) using shape variables was carried out independently for sexual and asexual individuals from localities where they are found in sympatry. We thereafter extracted the centroids on PCA plot for each group of individuals characterized by the sampling location, as proposed by Wang et al. (2010). Finally, centroids’ PCA coordinates of asexual individuals were aligned with sexual ones using a Procrustean superimposition approach (Gower, 1971; Legendre & Legendre, 1998). The degree of concordance between the ordination results of the two biotypes was assessed with a correlation-like statistic *r* PROTEST, derived from the symmetric Procrustes sum of squares, where 1 is perfect concordance and 0 the complete absence of concordance (Jackson, 1995). Significance of the obtained *r* correlation-like statistic was tested by permutation using 999 randomizations, as described by Jackson (1995), and under the null hypothesis that there is no concordance between sexual and asexual shape changes among environments.

#### Within-site shape variation

The extent of shape variation within sampling sites was used to assess the extent of niche use in natural localities. We performed analyses of multivariate homogeneity of group dispersions (Anderson, 2006), a multivariate analogue of Levene’s test for homogeneity of variance, to compared within-site variation for individuals in both controlled and natural conditions. Groups of individuals were defined according to their sampling location (specific natural localities vs common garden) and their biotypes (sexual vs asexual). Groups of asexual individuals were also specified according to their lineage to emphasize the genetic uniformity of individuals within a given lineage. For each group, Euclidean distances between individuals and centroid were computed. The mean distance to centroid of each individual within a given environment was then subjected to a one-way ANOVA if normality and homoscedasticity of the data were observed. Otherwise, data were log-transformed before ANOVA analyses. Post-hoc corrections were performed for cases of multiple comparisons, using Tukey’s “honest significant difference” (HSD) test.

All statistical analyses were computed with the statistical programming environment R version 3.2.4. Specifically, we used the *geomorph* package version 3.0.2 (Adams & Otárola-Castillo, 2013) for standard geometric morphometric analyses and the *vegan* package version 2.3-2 (Oksanen et al., 2015) for multivariate analyses.

## Results

### Measurement error

Procrustes ANOVA analyses revealed that shape variations are not different between the landmark positioning (*P* = 0.999) or imaging (*P* = 0.168) sessions. Moreover, variation due to inter-individual differences was much higher than within-individual variation due to landmark positioning (*R*^2^ = 97.31%, *P* = 0.001) or individual digitizing (*R*^2^ = 87.99%, *P* = 0.001). These results indicated that shape differences detected between two different randomly selected individuals were higher than differences due to landmark positioning or imaging of one individual. Measurement error was therefore considered as low since inter-individual variation was expected to be higher than the proportion of variation due to fish manipulation or landmark positioning.

### Common garden experiments

**Figure 2:**
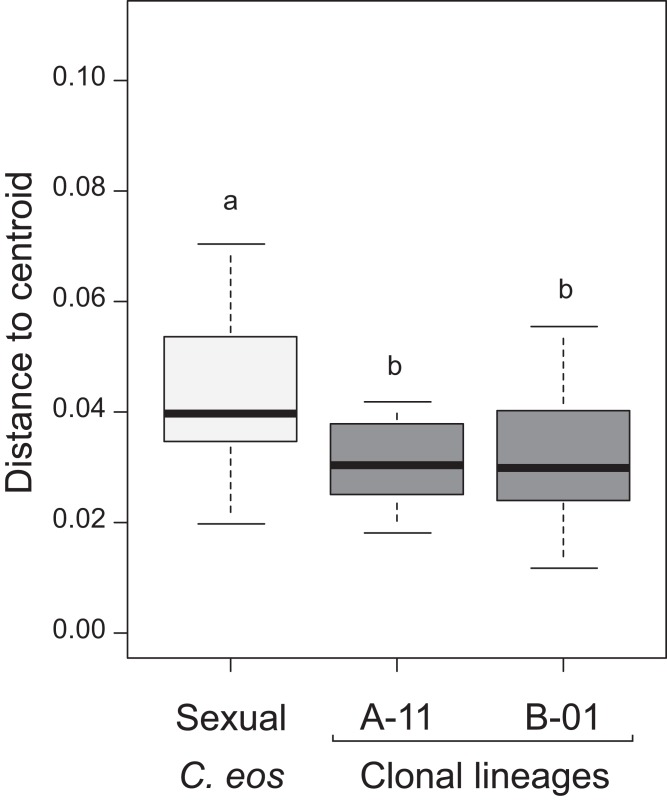
Morphological variation within common garden. Results of ANOVA analysis of groups’ dispersion. Pairwise comparisons sharing different lowercase letters are significantly different (*P* < 0.012, Tukey HSD).

Individuals reared in common garden (*n* = 63) were composed of: 28 sexual *C. eos* and 11 individuals of the A-11 lineage from the RI-2 stream and 24 individuals of the B-01 lineage from the AS-16 lake. Analysis of variation of individuals in controlled conditions revealed that both clonal lineages displayed lower morphological variation than the group of sexual individuals (*P* < 0.012, Tukey HSD post-hoc, Fig. 2). Shape variation was still detected among genetically identical individuals (within a given lineage) reared in a homogeneous environment, indicating that stochastic factors and/or social interactions might influence phenotypic variation. Partition of shape variation revealed significant shape differences between asexual and sexual individuals (*R*_a_^2^ = 16.74%, *P* = 0.001), as well as between the two asexual lineages, but at a lower extent (*R*_a_^2^ = 6.10%, *P* = 0.010), confirming the genetic effect on phenotypic variation.

**Figure 3:**
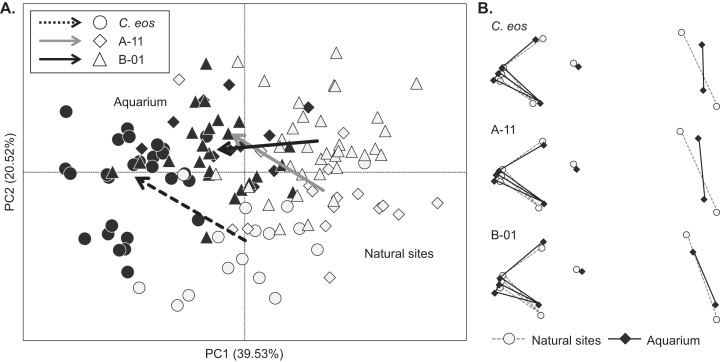
Morphological changes due to the transfer from natural to controlled conditions. (A) Principal component analysis with individuals from natural conditions (open symbols) and those reared in common garden (filled symbols). Arrows represent the magnitude and direction of morphological changes for *C. eos* (dotted black), lineage A-11 (solid gray) and lineage B-01 (solid black). (B) Landmarks of the mean shape of natural (open symbols) and controlled (filled symbols) conditions for each group. Morphological changes were magnified tree times to show shape differences.

When compared to individuals from their respective sampling sites in natural conditions, transfer to controlled conditions induced significant shape changes for both *C. eos* and clonal individuals (*R*_a_^2^ = 14.97%, *P* = 0.001). However, sexual *C. eos* and both clonal lineages did not respond to the transfer in the same way (Groups × Environments interaction: *R*_a_^2^ = 2.74%, *P* = 0.001). We compared the differences in biotypes response to the transfer from one environment to another both in term of “path distance magnitude of shape changes” and “direction of changes.” The proportion of shape variation explained by the transfer was of the same extent for *C. eos* individuals (*R*_a_^2^ = 22.36%, *P* = 0.001) and the two clonal lineages (*R*_a_^2^ = 24.31% and 19.51%, *P* = 0.001 for A-11 and B-01, respectively), and trajectory analyses confirmed that no difference in shape changes was detected in term of path distance magnitude (*P* > 0.204) among the three groups (Fig. 3A). In contrast, shape trajectory differed in term of direction of changes according to the type of sampling site: shape change trajectories of *C. eos* and lineage A-11 individuals, that came from the same stream, displayed the same direction (*P* = 0.704), whereas a non-parallel shape trajectory was observed for lineage B-01 individuals that were sampled from a lake, compared to the two other groups (*P* < 0.003; Fig. 3A).

Similar results could also be observed in landmarks positions. Indeed, we observed that transfer from natural to controlled conditions induced a similar change in the shape of the mouth’s angle for the three groups (landmarks #1 to #6, Fig. 3B), suggesting that identical feeding mode resulted in similar morphological changes. In contrast, a more pronounced shift was observed at landmarks #7 and #8 for *C. eos* and lineage A-11 individuals sampled from the same stream compared to lineage B-01 individuals sampled from a lake (Fig. 3B). This difference involved changes at the level of the operculum and the pectoral fin that occurred according to sampled sites but not to biotype.

### *C. eos* and asexual *C. eos-neogaeus* in natural conditions

We sampled a total of 602 individuals (290 *C. eos* and 312 clonal individuals) from 20 field sites for geometric morphometric analyses (Table 1). The sexual *C. eos* was found in allopatry in 10 localities while both sexual and clonal individuals were found in sympatry in the remaining 10 sampled sites (Table 1). For each of the 10 sites where sexual and clonal individuals were found in sympatry, biotypes occurred in equal proportion (*χ*^2^, *P* > 0.265 after Holm (1979) correction for multiple comparisons), except in site SF-14 where more hybrid individuals were sampled compared to *C. eos* individuals (*χ*^2^, *P* = 0.018 after Holm correction for multiple comparisons).

**Figure 4:**
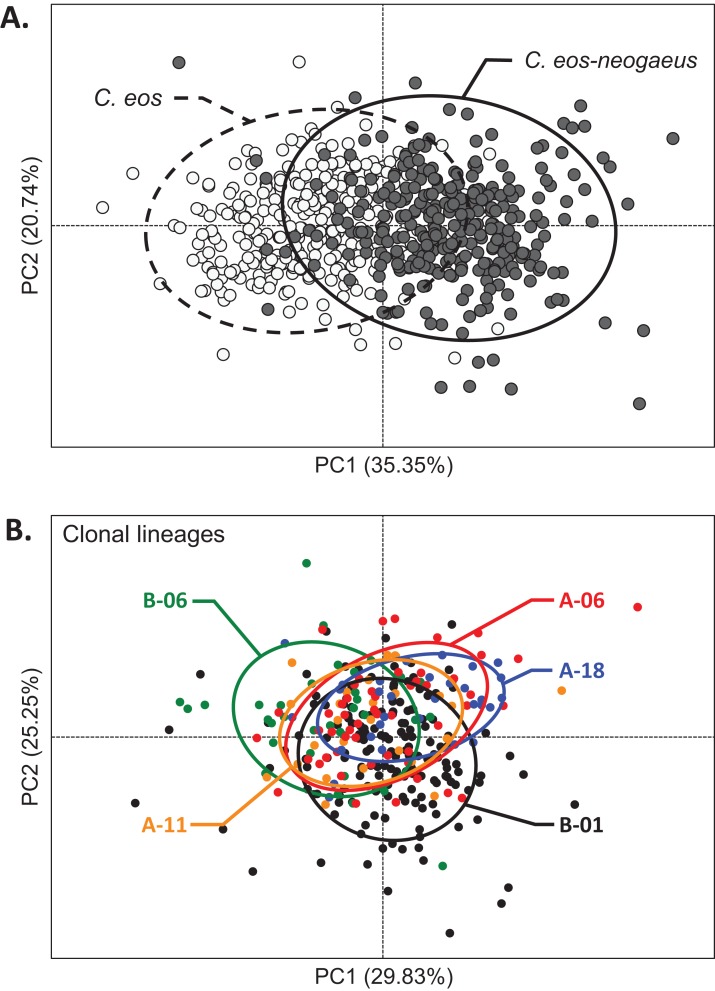
Shape variation of individuals from natural populations. (A) PCA scatter plot with 95% confidence interval ellipses for *C. eos* (open circles and dotted line) and the five clonal *C. eos-neogaeus* lineages (filled circles and solid line). (B) PCA scatter plot with 95% confidence interval ellipse for the five clonal lineages; each color corresponds to a different lineage.

A significant difference in shape was detected between *C. eos* and clonal hybrid (*R*_a_^2^ = 19.03%; *P* = 0.001). However, similar extent of morphological variation was observed between biotypes. First, sexual *C. eos* and clonal individuals displayed, overall, the same dispersion to group centroid (*F*_1,600_ = 0.8635; *P* = 0.383), and there was an important overlap between sexual and asexual individuals (Fig. 4A). Interestingly, the five lineages shared on average the same morphospace (Fig. 4B) and they all displayed similar extent of morphological variation in natural environments (*F*_4,307_ = 1.4084; *P* = 0.231). Second, the same extent of shape differences was detected among sampled sites for both sexual (*R*_a_^2^ = 10.26%, *P* = 0.001) and asexual (*R*_a_^2^ = 11.26%, *P* = 0.001) individuals. These results were obtained by taking the asexual genotype at each site as conditioning matrix for both analyses involving sexual and asexual individuals, and highlighted therefore the propensity of clonal individuals to display as much phenotypic variation as the sexual species despite their absence of genetic variation among individuals.

### *C. eos* and *C. eos-neogaeus* in sympatry

**Figure 5:**
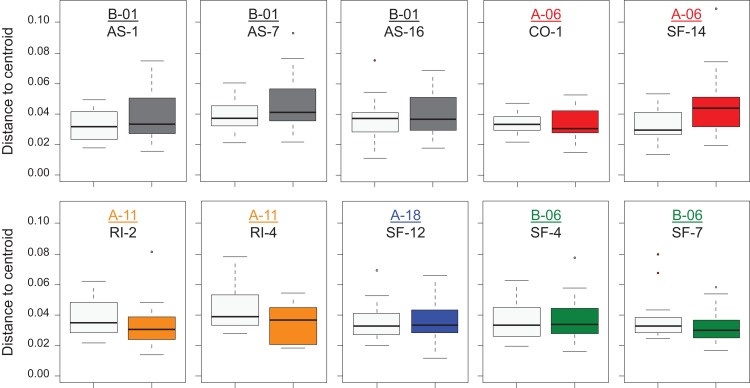
Intra-site shape variation in sympatric localities. Boxplot with median, quartiles and range of individuals distance to centroid to assess *C. eos* (open boxes) and asexual (filled boxes) individuals dispersion within each site. Lineages (colored and underlined) and sites code are according to Table 1.

To take into account environmental differences among sites, comparisons of sexual and clonal individuals were restricted to the 10 localities where both biotypes were in sympatry. Once again, both sexual and asexual individuals displayed the same total variation (distance to centroid analysis: *F*_1,442_ = 0.8539, *P* = 0.357). Furthermore, the same extent of morphological variation was observed for both sexual *C. eos* and clonal individuals within a given site (*P* > 0.369, Tukey HSD post hoc; Fig. 5). This result contrasts with the measured variation within common garden environment where *C. eos* individuals displayed higher variation than clonal ones (Fig. 2). Interestingly, the comparison of individuals reared in controlled conditions with those from their respective sampled site revealed that individuals displayed the same variation in controlled and natural conditions for sexual *C. eos* (*F*_1, 42_ = 1.172, *P* = 0.285) and lineage A-11 (*F*_1, 26_ = 0.314, *P* = 0.580) from the same sampled stream. However, a significant reduction of morphological variation was observed for clonal individuals of lineage B-01 from the lake-type environment when reared in an homogeneous environment (*F*_1, 57_ = 4.564, *P* = 0.037).

Similarly, the two biotypes within a given site appeared to be as different as in a homogeneous environment, as shape difference in term of path distance magnitude were not significantly different from that observed in common garden for six out of the 10 sites (AS-7, RI-2, RI-4, SF-4, SF-12, and SF-14; *P* > 0.084). However, shape differences between *C. eos* and clonal individuals appeared to be greater than those observed in common garden for the four remaining sites (AS-1, AS-16, CO-1, and SF-7; *P* < 0.021).

Interestingly, using Procrustes superimposition analysis, we detected no concordance among environments between shape changes of *C. eos* with those of clonal individuals when considering all sites where they were found in sympatry as well as common garden (*r* PROTEST = 0.677, *P* = 0.089). Nonetheless, significant concordance between sexual and asexual morphospaces was detected when considering only the six sites where *C. eos* and asexual individuals were as different as in common garden (AS-7, RI-2, RI-4, SF-4, SF-12, and SF-14; *r* = 0.879, *P* = 0.001). These results suggest that clones can occupy the same range of environments than sexual individuals. Alternatively, no concordance was identified for the four remaining sites (AS-1, AS-16, CO-1, and SF-7; *r* = 0.893, *P* = 0.091), suggesting that asexual and sexual individuals can occupy distinct environments at a given site. However, such differences in *C. eos* and asexual individuals morphospaces could not be explained by hybrid genotypes, nor the type of environments, as the same lineages were found in the two categories of sites, and both in lakes and stream type environments (Table 1).

## Discussion

In this study, we used a trait-based ecology approach exploiting trophic and locomotive structures to compare the extent of the realized niches between asexual and sexual individuals across different environments. First, common garden experiments highlighted the capacity of both sexual and asexual individuals to cope with environmental heterogeneity via phenotypic plasticity. Then, the partition of morphological variation in natural conditions showed that plasticity promotes niche diversification in genetically identical individuals, allowing them to occupy a range of environmental conditions similar to that of sexual individuals.

### Sources of phenotypic variation

Our results underlined different sources of phenotypic variation, including genetic, environmental, and stochastic factors. First, the influence of genetic variation on phenotype was confirmed by the higher morphological variation for sexual compared to clonal individuals detected within controlled conditions (Case & Taper, 1986; Evans et al., 2016). The different genomic composition of sexual *C. eos* and clonal *C. eos-neogaeus* also resulted in shape differences both in controlled and natural environments. This is in accordance with previous studies comparing the morphology of both biotypes (Doeringsfeld et al., 2004; Schlosser et al., 1998). Contrary to these studies, however, an important overlap was observed between sexual *C. eos* and clones’ morphospace indicating that, according to trait analyzed in this study, the shape of individuals of different biotypes could be more similar than for individuals of a given biotype. Although genetically variable, morphological differences detected between sexual individuals reared in common garden and those from their relative natural sites could not be attributed to different adaptations since comparisons were made with individuals that all originated from the same population.

Second, we observed, for both sexual and clonal individuals from a given site, the same trajectory of morphological changes following the transfer from natural to controlled conditions. The artificial feeding mode has resulted in a similar change at the level of mouth angle for both biotypes. Moreover, the absence of water velocity within the aquariums caused similar changes at the level of the operculum and the pectoral fin for fish regardless of biotypes. This convergence in trajectories in response to the same environmental conditions suggests that both sexual *C. eos* and clonal *C. eos-neogaeus* display the same capacity for phenotypic plasticity, a key component to promote population divergence and allow the persistence of lineages (Pfennig et al., 2010). Furthermore, phenotypic response of clonal *C. eos-neogaeus* to environmental heterogeneity is consistent with previous studies that reported distinct epigenetic profiles according to environmental conditions (Leung, Breton & Angers, 2016; Massicotte & Angers, 2012).

Altogether, these results highlight the predominant role of the environment on the measured morphological variation within a given biotype. This also confirms that the morphology of mouth and fins measured in this study represents a good *proxy* of *Chrosomus* spp. feeding ecology and locomotion, respectively.

Finally, even when controlling for genetic and environment, shape differences were detected among genetically identical individuals within a homogeneous environment. This result is among the first to show morphological variation among clonal individuals in multiple fish lineages, while it has been shown on other phenotypic traits like behavior (Bierbach, Laskowski & Wolf, 2017; Makowicz et al., 2016). Such phenotypic differences might be due to individuals’ interactions (Bolnick et al., 2011; Dall et al., 2012) and/or to stochastic factors (Bierbach, Laskowski & Wolf, 2017), for instance because of random epigenetic changes (Leung, Breton & Angers, 2016). An alternative hypothesis is that the observed variation may also be a remnant of maternal effects or heterogeneous environmental conditions on the early development stages, as larvae were born in natural environments.

### Distribution of morphological variation in natural populations

Comparisons of individuals from natural environments revealed that sexual and clonal individuals displayed similar extent of morphological variation. We detected significant concordance between sexual and asexual morphospace with the Procrustes superimposition analysis for six of the ten sites where the two biotypes were found in sympatry. This indicates that sexual and asexual individuals displayed similar morphological trajectories among sampled sites in response to the different environmental conditions. In addition, we also observed that asexual individuals could be as variable as sexual ones in natural environments despite their genetic uniformity, which is consistent with previous studies (Doeringsfeld et al., 2004; Schlosser et al., 1998). However, results from natural environments contrasted with those from common garden experiments, where clonal individuals displayed significantly lower levels of morphological variation (i.e., a consistent phenotype was observed). We can interpret the high intra-site variation of clones in natural conditions with two non-mutually exclusive hypotheses: (1) asexual individuals might be confronted to a larger environmental heterogeneity within a given site, or (2) they display higher sensitivity to environmental signals, compared to sexual individuals.

Concerning hypothesis #1, our results showed that in controlled conditions, sexual individuals exhibited a higher phenotypic variation than clones due to their genetic variability. Therefore, the higher morphological variation of clones in natural conditions but of the same extent than their sexual counterparts could illustrate a larger range of ecological niches for clonal lineages. Alternatively, the similar extent of morphological variation for sexual individuals in controlled conditions and natural sites suggests that they occupied less diversified niches than their asexual counterpart in nature, otherwise, sexual individuals were expected to be morphologically more variable than clonal ones, as observed in common garden experiment where both biotypes were constrained for the same niche. These results are contrary to what is expected under a model based on the assumption that genetic differences are translated into ecological differences (Case & Taper, 1986), strengthening therefore the hypothesis that alternatives to genetic variation could result in phenotypic variation and, thus, niche diversification (Hanley, Bolger & Case, 1994).

Alternatively, concerning hypothesis #2, both sexual and asexual individuals can occupy the same range of environmental conditions, but a higher sensitivity of asexual individuals to environmental signals would result in higher morphological variation. This hypothesis is consistent with a previous study performed on another phenotypic trait: the dental formulae (Leung et al., 2017). Indeed, a relative stability of dental formulae was reported in multiple fish species including the parental species *C. eos* (Eastman & Underhill, 1973). This contrasted with the high variation detected for the asexual *C. eos-neogaeus* (Leung et al., 2017). The higher variation in clones could be explained by their higher sensitivity to environmental signals triggering alternative developmental pathways, thus contrasting with *C. eos*’ higher canalization for the same phenotype (Leung et al., 2017).

The two hypotheses proposed above are, however, not mutually exclusive and this can also be illustrated with our results. For six out of the 10 sites, the same extent of morphological differences were measured between sexual and asexual individuals as in common garden and a concordance of the two biotypes’ morphospaces was observed. These results could indicate that sexual and asexual individuals used the same ecological niches, but clones were found to be as variable as sexual individuals because of their higher sensibility to environmental cues. By contrast, for the four remaining sites, morphological differences between sexual *C. eos* and asexual individuals were higher than the differences observed in a homogeneous environment. Moreover, these four sites were characterized by an absence of concordance between the morphospaces of sexual and asexual individuals. These results suggest that sexual and clonal individuals used distinct ecological niches at these sites and could therefore support a niche diversification hypothesis. Coexistence of sexual and sperm-dependent clonal species is a challenging puzzle as demographic advantage of asexual organisms may drive them to extinction if they outcompete and replace their sexual host (Kokko, Heubel & Rankin, 2008; Lehtonen et al., 2013; Leung & Angers, 2018). Niche separation has then been proposed as a means to reduce competition between asexual organisms and their sexual hosts, thus explaining the coexistence of sexual and asexual organisms (Gray & Weeks, 2001; Schley, Doncaster & Sluckin, 2004; Schlosser et al., 1998; Schlupp, 2005; Vrijenhoek, 1994; Weeks et al., 1992). For instance, even in the absence of male preference for sexually-reproducing females, the segregation of sexual and asexual individuals in the field may result in an indirect discrimination toward asexual females, which may have less chance to be inseminated than sexual females (Booij & Guldemond, 1984).

### Ecological benefits of phenotypic plasticity

In the absence of genetic variation, we argue phenotypic plasticity is crucial for the persistence of asexual organisms. This is particularly well exemplified in sperm-dependent clonal organisms as, despite their genetic uniformity, they have to coexist with a related sexual species that can stand on genetic variation (in addition to plasticity) to cope with environmental heterogeneity. The phenotypic variation observed in the sexual host in different environments can then be used as a comparative reference to assess the acclimation of asexual individuals and illustrate the role of plasticity in coping with environmental heterogeneity.

Our results highlight the surprising capacity of asexual individuals to modulate their range of effective ecological niches, which may explain their widespread distribution. Indeed, this niche diversification of clonal individuals may help to avoid niche overlap and reduce competition with the sexual form at a local scale. The two biotypes are also known to display distinct parasite load (Mee & Rowe, 2006) and morphology (Doeringsfeld et al., 2004; Schlosser et al., 1998), suggesting they indeed use distinct niches. Furthermore, significant shape differences between sexual and asexual individuals were observed both within controlled and natural environments, suggesting that coexisting biotypes responded differently to a given environment signal, or, on average, they exploited different niches within a given site.

## Conclusion

In conclusion, this study highlighted the propensity of asexual organisms, despite their genetic uniformity, to be morphologically as variable as a sexual species. Furthermore, our results indicated that clones can efficiently use different niches and may evolve in a range of environmental conditions comparable to those of a sexual species. This study therefore underlines the importance of factors other than genetic variability, like epigenetic processes promoting phenotypic plasticity (Angers, Castonguay & Massicotte, 2010; Castonguay & Angers, 2012), for coping with environmental heterogeneity.

## Supplemental Information

10.7717/peerj.5896/supp-1 Supplemental Information 1 Two-dimensional morphometric raw data. For each individuals, biotype, lineage and sampled site are identified according to Table 1; CG = Common Garden.

## Additional Information and Declarations

### Field Study Permissions

The following information was supplied relating to field study approvals (i.e., approving body and any reference numbers): Sampling permits were provided by the Quebec Ministry of Natural Resources and Wildlife (MRNF).

### Data Availability

The following information was supplied regarding data availability: The two-dimensional morphometric raw data are available in Table S1.
